# Comprehensive Evaluation and Optimization of Amplicon Library Preparation Methods for High-Throughput Antibody Sequencing

**DOI:** 10.1371/journal.pone.0096727

**Published:** 2014-05-08

**Authors:** Ulrike Menzel, Victor Greiff, Tarik A. Khan, Ulrike Haessler, Ina Hellmann, Simon Friedensohn, Skylar C. Cook, Mark Pogson, Sai T. Reddy

**Affiliations:** Department of Biosystems Science and Engineering, ETH Zürich, Basel, Switzerland; New York University, United States of America

## Abstract

High-throughput sequencing (HTS) of antibody repertoire libraries has become a powerful tool in the field of systems immunology. However, numerous sources of bias in HTS workflows may affect the obtained antibody repertoire data. A crucial step in antibody library preparation is the addition of short platform-specific nucleotide adapter sequences. As of yet, the impact of the method of adapter addition on experimental library preparation and the resulting antibody repertoire HTS datasets has not been thoroughly investigated. Therefore, we compared three standard library preparation methods by performing Illumina HTS on antibody variable heavy genes from murine antibody-secreting cells. Clonal overlap and rank statistics demonstrated that the investigated methods produced equivalent HTS datasets. PCR-based methods were experimentally superior to ligation with respect to speed, efficiency, and practicality. Finally, using a two-step PCR based method we established a protocol for antibody repertoire library generation, beginning from inputs as low as 1 ng of total RNA. In summary, this study represents a major advance towards a standardized experimental framework for antibody HTS, thus opening up the potential for systems-based, cross-experiment meta-analyses of antibody repertoires.

## Introduction

High-throughput sequencing (HTS) of antibody repertoires offers the potential to study the humoral immune system in a quantitative and systems-based approach [1]–[4]. However, preceding HTS are many experimental steps in the multi-component library preparation, which are prone to biases and errors, and thus may substantially decrease the accuracy of the HTS delivered antibody repertoire [5]. These biases and errors are related to choice of nucleic acid material [6], PCR protocol variations [7]–[14], primers needed for specific amplification of antibody genes [15], [16], and multiplexed barcoding [17], [18]. Therefore, performing comprehensive analyses and establishing detailed experimental and bioinformatics methods has become very valuable for advancing HTS in systems biology research [3], [5], [10], [11], [19]–[22].

**Figure 1:**
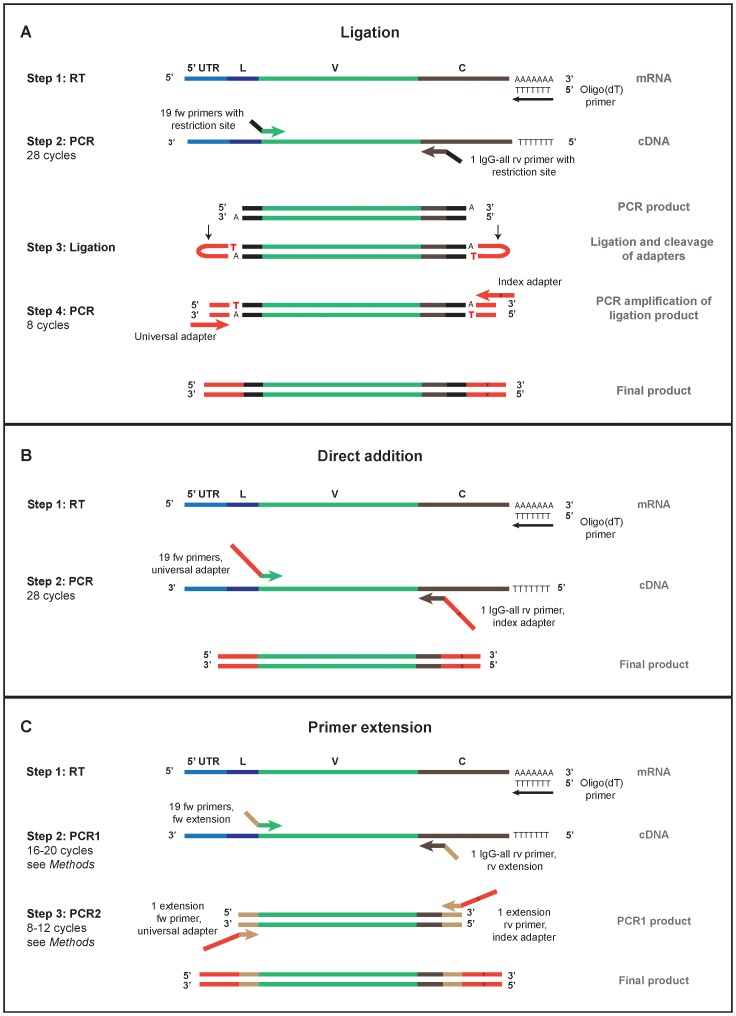
Overview of the different methods used for adapter addition to antibody variable heavy chain amplicon libraries. All methods required the reverse transcription of antibody mRNA into cDNA (step 1), which served as template for the following IgG gene-specific amplification by PCR. (A) The ligation method required a pre-amplified library as starting material, with a 3′ A-overhang added by the Taq DNA Polymerase (step 2). The stem-loop adapters containing a 5′ T-overhang were then attached in an enzymatic ligation reaction and cleaved in order to create a double-stranded form (step 3) that served as template for a final amplification step (step 4) in which the full-length Illumina TruSeq universal and index adapter sequences were incorporated into the library. (B) The direct addition method combined antibody library amplification and sequencing adapter addition into one PCR step (step 2) by attaching the Illumina adapter sequences 5′ of the gene-specific primers used for library preparation. (C) The primer extension method incorporated a GC-rich overhang into the library in PCR1 (step 2). This resulted in uniformly high amplification in a second PCR by using primers specific for the GC-rich overhang and containing the full-length Illumina sequencing adapters (step 3). UTR: untranslated region, L: leader sequence, V: variable region, C: constant region, RT: reverse transcription, fw: forward, rv: reverse, x: barcode/index allowing multiplexed sequencing runs.

One essential component of all amplicon library preparation methods for HTS is the addition of sequencing adapters. To date, the impact of adapter addition methods on antibody HTS has not been thoroughly determined. Adapters are dual-purpose, platform-specific oligonucleotide sequences required for nearly all HTS technologies (e.g., Illumina, 454, Ion Torrent, Pacific Biosciences, SOLiD). On the Illumina platform, they are essential to the sequencing biochemistry, enabling flow cell binding, cluster generation, and reaction priming. They also permit indexing of samples to perform efficient multiplexed sequencing runs. Adapters are attached to the 5′ and 3′ ends of the genetic fragments of interest to yield the sequencing-ready library. Commonly used methods are based on ligation or PCR-addition of the sequencing adapters. In the ligation method, the antibody libraries are first amplified by PCR using a primer set specific for the targeted variable heavy or light chain regions. Subsequently, double-stranded oligonucleotides partly containing the adapter sequences are attached by ligation and then followed by a low-cycle PCR amplification step (i.e., 4–8 cycles), which completes the addition of full-length adapter sequences [16], [23], [24]. Recently, PCR-based methods have been introduced for adapter addition [15], [25], [26] in either a one-step (direct addition, DA) or two-step (primer extension, PE) PCR reaction (Fig. 1).

This study investigated the influence of methods of adapter addition on both the PCR and sequencing level in terms of experimental efficiency, practicality, and impact on resulting HTS datasets. Using the same starting total RNA source isolated from pooled antibody-secreting cells (ASCs) from immunized mice, antibody heavy chain variable region (V_H_) libraries were prepared for Illumina-based HTS using ligation-, DA-, or PE-based adapter addition. Additionally, using the PE method, a titration of total RNA was conducted to determine a potential lower limit of total RNA input. HTS datasets were compared based on reliably detected clones [27], using the amino acid sequence of the complementarity determining region 3 (CDR3) as a clonal identifier. Reliability was measured as the simultaneous presence of CDR3 sequences in the compared datasets. The comparison of ligation, DA, and PE in terms of yield and repertoire composition provided the following insights: (i) All methods (Ligation/DA/PE) yielded highly comparable HTS datasets–the similarity in antibody repertoire representation was in the range of technical replicates. (ii) However, based on experimental library preparation criteria, DA and PE were superior to ligation in terms of either practicality (DA) or yield and efficiency (PE). (iii) PE was validated and optimized as the method of choice in cases of limited RNA starting material, as we were able to successfully generate HTS libraries with as little as 1 ng of total RNA, which is critical for immunological studies that focus on rare B-cell populations. The here established set of experimental and statistical guidelines for both library preparation and data analysis represents an important move towards a standardized experimental framework for antibody HTS, which will be valuable for cross-study and cross-laboratory comparisons, thereby advancing the field of systems immunology [3], [19].

## Materials and Methods

### Animal Experiments and Cell Isolation

All animal experiments were performed under the guidelines and protocols approved by the Basel-Stadt cantonal veterinary office (Basel-Stadt Kantonales Veterinäramt Tierversuchsbewilligung #2582). Nine female BALB/c mice (Charles Rivers Laboratories) 8–10 weeks old were housed under specific pathogen-free conditions and were maintained on a normal chow diet. Mice were immunized with 50 µg alum-precipitated chicken gamma globulin (CGG) conjugated to 4-hydroxy-3-nitrophenylacetyl (NP, NP-CGG from BioCat) and were sacrificed 14 days post-immunization. Spleens and bone marrow (from femurs and tibia) were harvested and antibody-secreting CD138-positive cells were enriched as previously described [23].

### Preparation of Antibody Libraries for High-throughput Sequencing

Total RNA extraction from the pooled ASC population was performed using the TRIzol Plus RNA Purification Kit (Life Technologies) according to the manufacturer’s protocol. RNA concentration was determined on a Nanodrop 2000c Spectrophotometer and RNA integrity and concentration were evaluated on a Bioanalyzer 2100 (Agilent). Isolated total RNA was homogeneously mixed, aliquoted, and stored at −80°C. Next, first-strand cDNA was synthesized with Maxima Reverse Transcriptase (Fermentas) using 500 ng total RNA and Oligo(dT) primers (Thermo Scientific) following the manufacturer’s protocol. cDNA from multiple reactions was pooled, mixed, and stored in aliquots at −80°C to serve as PCR template for all tested methods. PCR amplification of the variable heavy IgG genes was performed using a set of 19 forward primers with the gene-specific regions annealing to framework 1 of the VDJ-region [28] and a reverse primer with the gene-specific region binding to the IgG constant region 1 (5′ CARKGGATRRRCHGATGGGG 3′) [27]. PCR was performed with Taq DNA polymerase (NEB) in a reaction volume of 50 µl using 2 µl of the cDNA product as template and setting following conditions as standard [23]: 95°C for 3 min; 4 cycles of 95°C for 30 sec, 50°C for 30 sec, 68°C for 1 min; 4 cycles of 95°C for 30 sec, 55°C for 30 sec, 68°C for 1 min; 20 cycles of 95°C for 30 sec, 63°C for 30 sec, 68°C for 1 min; 68°C for 7 min; 4°C storage. PCR cleanup was performed to reduce the sample volume of parallel reactions, followed by gel-excision and purification of bands of appropriate size (550–600 bp depending on overhang lengths) from 1% agarose gels.

Ligation method: Libraries were generated using the aforementioned gene-specific primer set and PCR conditions, with the distinction that primers contained 5′ restriction sites (Fig. 1A) as previously described [29]. Parallel reactions were run to obtain ≈1 µg of gel-purified DNA library, which is the recommended minimum input for the adapter ligation kit used (NEBNext Multiplex Oligos for Illumina Kit, New England Biolabs, NEB). Adapter ligation was performed following the manufacturer’s instructions (NEB). Gel-purification was performed as the very last step in order to obtain a clean library for HTS (Fig. S1A in File S1).

Direct addition (DA) method: The Illumina TruSeq universal adapter constituted the 5′ portion of the forward primers, while the IgG-reverse primer contained the Illumina index adapter, which resulted in direct addition of Illumina adapter sequences to the PCR products (Fig. 1B, Table S3 in File S1). Ten PCR reactions were run in parallel with the standard PCR conditions mentioned above, which is equivalent to 500 ng total RNA input. Two replicate libraries using the direct addition method were prepared as detailed above (DA1/DA2). It was also tested whether 400 and 200 ng RNA were sufficient for library generation. Fresh cDNA was prepared with calculated RNA amounts and used as PCR template (10 parallel reactions). Prepared libraries were concentrated and purified as described above (Fig. S1B/C in File S1).

Primer extension method: Pre-amplification was achieved in a first round PCR using the gene-specific primer regions containing a 5′ GC-rich overhang (Fig. 1C, Table S3 in File S1). Different cycle numbers were tested. Two samples were prepared with 8 cycles (PE1/2) while one sample (PE3) was prepared with 12 cycles in the last annealing temperature step (95°C for 30 sec, 63°C for 30 sec, 68°C for 1 min) of the standard PCR conditions in PCR1 resulting in 16 or 20 total cycles, respectively. All samples were subjected to PCR cleanup. Products of appropriate size (≈500 bp) were purified from a 1% agarose gel and the entire product was used as template for the second round PCR, which used only one forward and one reverse primer resulting in the addition of full-length universal and index adapter sequences to the library. PCR conditions of the second round PCR (PCR2) were either 95°C for 3 min; 5 cycles of 95°C for 30 sec, 40°C for 30 sec, 68°C for 1 min; 7 cycles of 95°C for 30 sec, 65°C for 30 sec, 68°C for 1 min; 68°C for 7 min, 4°C storage (for PE1, 28 overall cycles) or 95°C for 3 min; 3 cycles of 95°C for 30 sec, 40°C for 30 sec, 68°C for 1 min; 5 cycles of 95°C for 30 sec, 65°C for 30 sec, 68°C for 1 min; 68°C for 7 min, 4°C storage (for PE2, 24 overall cycles and PE3, 28 overall PCR cycles, Fig. S1D in File S1). PCR2 was performed in 5 parallel reactions.

For the RNA titration analysis, PE was performed using 100 ng and 50 ng total input RNA following the same PCR conditions as sample PE3 and the final product was gel-purified using the QIAquick Gel Extraction kit (Qiagen). PCR conditions of low RNA samples (10 ng, 5 ng, 1 ng) were extended by two additional cycles in the last annealing temperature step of PCR2 (7 cycles of 95°C for 30 sec, 65°C for 30 sec; 30 total cycles) and the final product (≈600 bp) was gel-purified (Fig. S1E in File S1) using the MinElute Gel Extraction kit (Qiagen).

Prior to HTS, all amplicon libraries were submitted for a final quality control step on a Bioanalyzer 2100 (Agilent). Only libraries giving a single clear peak of appropriate fragment length were submitted for Illumina HTS. Calculated amounts of sample DNA libraries were pooled to a 4 nM concentration for read equilibration of parallel multiplexed sequencing.

### Illumina Sequencing, Data Analysis, and Statistics

All samples were sequenced using the Illumina MiSeq platform with 2×250 bp paired-end reads. Raw data can be accessed from the European Nucleotide Archive (ENA Study Accession ERP004622). Forward and reverse reads were paired using PANDAseq with default parameters [30]. CDR3 and full length VDJ region annotation of successfully paired sequences was performed using ImMunoGeneTics (IMGT)/HighV-QUEST [31], [32]. For downstream analyses, sequences were pre-processed and reads only retained based on the following criteria: (i) both CDR3 and VDJ region could be detected by IMGT/HighV-QUEST; (ii) CDR3s were of minimal length of 4 amino acids; (iii) CDR3 and VDJ regions were present with a minimum abundance of 2. For VDJ comparative analyses, primer trimming was performed using the CLC Genomics Workbench. For all analyses, CDR3 and VDJ abundances were calculated based on occurrence of exact amino acid sequences (100% identity). For all HTS dataset comparisons, libraries DA1 and PE3 were used if not mentioned otherwise. Spearman’s rank correlation coefficient was regarded as significant if p<0.05.

### Software

Starting from IMGT/HighV-QUEST output, data analysis was performed using the R statistical programming environment [33]. Non-base R packages used for analyses were: ggplot2 [33], [34], ShortRead [35] and hexbin [36].

### Reliable Detection Analysis

**Figure 2:**
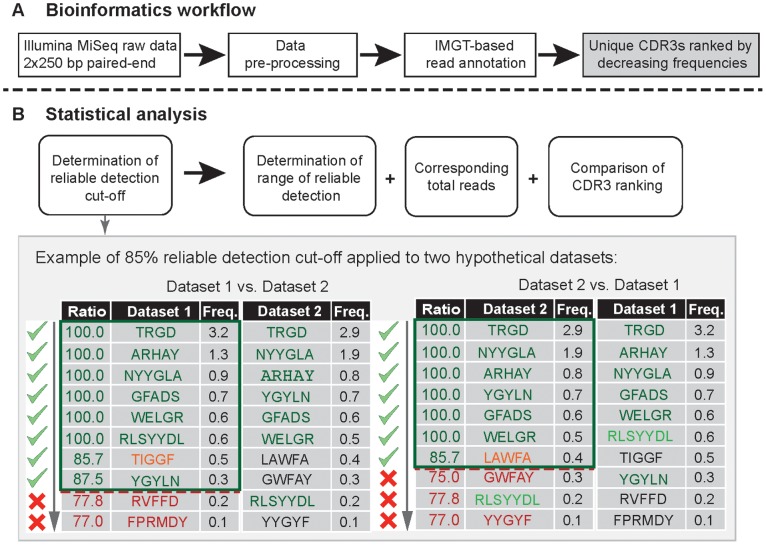
An overview of bioinformatics workflow and statistical analysis performed on antibody HTS datasets. (A) Bioinformatics steps following the HTS of antibody libraries and preceding the data analysis. Sequences were pre-processed and IMGT-annotated reads were filtered for (i) CDR3s of minimal length of 4 amino acids and (ii) CDR3 and VDJ regions present with a minimal abundance of 2 in order to exclude errors introduced during library preparation or HTS reaction. Abundances were calculated based on occurrence of exact amino acid sequences (100% identity, see *Methods*). (B) Statistical analysis detailing the principle of reliable detection as applied in this study. CDR3 clones were ranked in decreasing order of frequency and tested for simultaneous presence in the other dataset(s). The principle is demonstrated using two hypothetical datasets of 10 different (unique) CDR3s with an exemplary reliable detection cut-off of 85% (throughout this study 95% was used). Specifically, from each dataset a list of reliably detected clones was generated (clones in green box) by sequentially adding the highest frequency clones to a list, until the addition of the next clone would reduce the percentage of clones present in the other dataset(s) below the set threshold (marked by the red dashed line). All further clones of lower rank were not included in the list and therefore not reliably detected (red color). Orange indicates clones that are not present in the other dataset(s) but were nevertheless included in the list of reliably detected CDR3s via the 85% detection cut-off. The list of reliably detected CDR3s allowed downstream analyses such as the determination of the range of reliable detection (frequency and abundance ranges), the calculation of the percentage of total reads corresponding to the reliably detected CDR3s, and the comparison of CDR3 rankings.

Filtered and IMGT detected CDR3s were ranked in decreasing order of frequency and tested for simultaneous presence in the other dataset(s). We regarded all clones as “reliably detected” that belonged to the highest frequency set of clones with 95% of the contained clones present in the other dataset(s). Specifically, a list of reliably detected clones was generated from each dataset by sequentially adding the highest-frequency clones to the list, until the percentage of present clones fell below the set threshold of 95% (Fig. 2B).

## Results

In order to generate sufficient amounts of antibody RNA for comparisons of Illumina sequencing adapter addition methods, we collected and pooled splenic and bone marrow-derived ASCs of 9 mice 14 days after immunization with the common antigen NP-CGG. Total RNA was extracted, followed by first-strand cDNA synthesis. For all library preparation methods, we used an antibody variable heavy chain gene-specific primer set that binds to the first 20–22 nucleotides of framework region 1 (FR1, Table S3 in File S1) [28]. This ensured that differences among HTS datasets were solely attributable to the method of adapter addition used. The degenerate reverse primer was specific for the constant regions of all IgG gene subclasses (Fig. 1).

For the ligation method of adapter addition, the antibody library was generated using primers with overhangs containing restriction sites that offer the potential for library cloning (Fig. 1A) [29]. This DNA library served as input for the enzymatic ligation of the sequencing adapters using a commercial kit for Illumina adapter addition (NEB), which, according to the manufacturer’s recommendation, required a minimum starting material of 1 µg of DNA. By using Taq DNA polymerase, which adds a single-base non-templated adenine (A) overhang to the 3′ ends of the DNA product, the steps of end repair and A-tailing could be omitted. The adapters, in the form of a stem-loop, annealed via their free thymine-overhang to the adenine-overhang on the DNA product. They were ligated and subsequently cleaved, creating a double-stranded form needed for primer binding in the downstream PCR reaction, where the full-length universal and index sequences were added to the template.

The second tested method, DA, combined PCR amplification of IgG V_H_ genes and adapter addition into a single-step PCR reaction (Fig. 1B, Table S3 in File S1). The forward and reverse gene-specific primers were extended by inclusion of the full-length Illumina sequencing adapters. The forward primer set contained the universal adapter sequence, while the IgG reverse primer added the index adapter sequence. Using this method, amplicon libraries could be prepared and indexed for multiplexed sequencing in parallel in a single step by choosing differently indexed reverse primers for the PCR reaction [11]–[13], [15], [17], [18], [27].

Lastly, we tested a stepwise PCR variation, PE, in which the gene-specific regions of the oligonucleotides were extended with a GC-rich overhang (Fig. 1C, Table S3 in File S1). After an initial amplification with reduced cycle numbers, the amplified product was gel-purified and served as template in a second PCR step using primers containing the Illumina adapter sequence 5′ of the complementary overhang sequence; the rationale for this method was increased primer specificity and uniformly high amplification rates [15],[16],[24],[26].

### Amplification Efficiency Differed Greatly among Tested Adapter Addition Methods

First, we determined the differences between methods of adapter addition (Ligation/DA/PE) in terms of yield, amplification efficiency, and overall practicality. For the ligation method, we followed the manufacturer’s recommendation of a minimum DNA input of 1 µg in order to produce a library of sufficient size for HTS. However, with almost 4 µg of total RNA required to yield 1 µg of amplified DNA library, a much higher number of pooled parallel PCR reactions was necessary than for the other methods (76 total reactions, Table 1). Despite requiring more input and 8 additional amplification cycles (36 in total) for adapter-template enrichment, the amount of final adapter-ligated library that could be extracted was low (23 ng total extracted DNA library, Table 1). Of interest, using 500 ng RNA input produced about 100 ng of preamplified DNA library, which is 10-fold less than what is recommended (NEB). Tested ligation resulted in nearly undetectable amounts of library, which was insufficient for sequencing; hence the need for large amounts of RNA and a high number of parallel PCR reactions to generate a HTS library of sufficient size for the ligation reaction. In addition, the enzymatic ligation reaction is not fully efficient, further reducing HTS yield. Ligation was a straightforward protocol when used with Taq polymerase and required about 6–7 h of preparation time, including the time for V_H_ amplification. Economically, the ligation kit is affordable, but the additional effort to generate the greater amount of amplicon starting material combined with the continuous purchase of kits may in the long term result in substantial costs for frequent users.

**Table 1:** PCR and HTS statistics for ligation, DA, and PE.

	Library preparation results				HTS results					
Libraries	Input RNA (ng)	Number of parallelPCR reactions	PCR cycles	Library yield(ng)	Paired-end reads	Paired reads	% paired	Mean Phredscore#	IMGT detectedCDR3s	% of IMGT detected filtered CDR3s
**Ligation**	3,800	76	36	23	1,294,990	602,705	93	34.9	547,788	91
**DA1** *	500	10	28	20	3,570,976	1,712,361	96	35.8	1,633,160	95
**DA2** *	500	10	28	20	3,301,634	1,554,425	94	35.3	1,462,642	94
**PE1**	500	10	28	485	4,523,138	2,195,397	97	35.5	2,043,728	93
**PE2**	500	10	24	40	5,467,398	2,651,873	97	35.4	2,481,153	94
**PE3**	500	10	28	486	3,218,388	1,566,068	97	35.0	1,457,148	93
**PE–100 ng**	100	10	28	100	2,838,918	1,392,892	98	36.5	1,278,588	92
**PE–50 ng**	50	10	28	35	2,340,488	1,146,565	98	35.6	988,160	86
**PE–10 ng**	10	5	30	47	4,073,414	1,993,566	98	35.7	1,712,458	86
**PE–5 ng**	5	5	30	22	4,425,044	2,169,989	98	36.2	1,903,230	88
**PE–1 ng**	1	5	30	5	1,891,294	926,098	98	35.1	725,141	78

Display of performance differences of tested methods and conditions in terms of library preparation and HTS results. Please refer to the *Methods* section for library preparation details.

*DA1 and DA2 are technical replicates.

# Phred scores range between 0 and 40 and reflect the quality of Illumina base calling.

In contrast to the ligation method, DA readily allowed the generation of the desired antibody repertoire library using a more typical input of 500 ng total RNA [16], [37]–[39] (Fig. 1 and Fig. S1B in File S1). DA generated similar amounts of library as ligation (20 ng total library, Table 1), but required nearly 8 times less input RNA, consequently less parallel PCR reactions, and less PCR cycles (10 reactions, 28 cycles, Table 1). Since adapters were attached during the PCR reaction, close to 100% of the final product was HTS-ready, allowing for robust HTS library preparation [27]. DA was also tested with a panel of reduced RNA inputs to determine the lower limit of RNA needed for library preparation. When total RNA input was reduced to 400 ng and 200 ng, library amplifications resulted in faint bands on an agarose gel (Fig. S1C in File S1), which were neither extracted nor sequenced. To summarize, DA proved to be a fast single-step method (≈3 h preparation time) by combining both library generation and HTS preparation in one reaction step, and was therefore logistically the most practical of the three tested methods. The purchase of the long primers (19 forward plus indexed reverse primers) results in higher initial costs. However, these can be amortized in the long term, as the quantity of primers is sufficient for frequent reuse.

By virtue of a second PCR step, the PE method enables robust library amplification, even from small amounts of starting template (Fig. 1C, Table 1). Starting with ≈500 ng total RNA, PE yielded ≈25 times more library than DA and well over 25 times more library than ligation (considering the fact that only a fraction of the final ligation product was adapter-ligated). Additionally, we assessed the impact of altering and reducing PCR cycle numbers in the PE protocol. We reduced the cycle numbers from 12 to 8 cycles in the last annealing temperature step (16 total cycles) of PCR1 and tested either 12 (PE1–28 total cycles) or 8 (PE2–24 total cycles) PCR2 cycles (Table 1). By this, total cycle numbers were held constant for PE1 and PE3 (28 cycles) compared to the standard PCR protocol, but 4 cycles were shifted from PCR1 to PCR2, which should amplify without bias due to the use of only one forward and one reverse overhang-specific primer [15]. In sample PE2 the influence of reducing the total amount of PCR cycles from 28 to 24 was tested. We found that switching cycles between PCR steps did not influence the library yield (PE1); equal amounts of amplicon library (≈485 ng) were obtained with both PCR protocols (PE1 and PE3, Table 1, Fig. S1D in File S1). Reducing the number of PCR cycles to a total of 24 (PE2) led to an over 10-fold reduction in library yield, yet still yielded nearly twice the amount of library generated with DA (Table 1, Fig. S1D in File S1). The second PCR step resulted in intermediate preparation time (≈5 h) for the PE method compared to ligation and DA. Due to the shortened primer length, the cost of the PE method is substantially reduced compared to DA, with the per-reaction cost possibly lower than that of the ligation kit: the amounts of primer provided allow for many library generations, which suggests that this method is potentially the least expensive of the three (Table 2).

**Table 2:** The relative advantages and disadvantages of the three investigated adapter addition methods (Ligation/DA/PE) for antibody HTS.

Method	Advantage	Disadvantage
**Ligation**	Commercial kits are available No specificprimer design and purchase requiredStraightforward method due to Taq polymerasegenerating 3′A-overhangs Affordableprice per library	High RNA input required Requires highest number of total PCR cycles (Table 1) Longest preparation time (6 h) Requires polymerase that creates single-base 3′ adenine-overhangs Potentially higher costs for many libraries
**Direct addition**	Fast (3 h preparation time) Single-step method(Fig. 1A) Works well with moderate RNA amounts(≥500 ng, Table 1, Fig. S1A/B in File S1)Works in principle with all polymerases Quantityof primers allows for generation of many libraries	Not suitable for very small amounts of RNA (Table 1, Fig. S1A/B in File S1) Initial purchase of primers is expensive
**Primer extension**	Most efficient method (Table 1, Fig. 4, S1D andS7 in File S1) Suitable for small RNA samples(Table 1, Fig. S7 in File S1) Works in principlewith all polymerases Short primers reduceoverall costs and allow generation ofmany libraries (Table S3 in File S1)	Requires more time than DA (5 h)

We additionally set out to determine a lower limit of RNA input that would still allow generation of a HTS-ready library when using the PE method. For this, RNA was titrated over two orders of magnitude (100 ng, 50 ng, 10 ng, 5 ng, 1 ng) and libraries were prepared using the PCR conditions of PE3. The lowest three RNA samples (10 ng, 5 ng, 1 ng) were amplified with two additional cycles in the last amplification step of PCR2 (30 total cycles). We were able to show that even with as little as 1 ng of starting total RNA the PE method could produce a visible and gel-extractable band (Fig. S1E in File S1) yielding sufficient product for subsequent HTS (Table 1).

In summary, we compared two PCR-based methods (DA and PE) and the ligation method for adapter addition and found experimental advantages and drawbacks for each choice, which are summarized in Table 2.

### All Tested Methods of Adapter Addition Yielded Sequencing Datasets of High Quality

HTS of samples was performed on the Illumina MiSeq platform using 2×250 bp paired-end reads. HTS yielded an average of 3.4×10^6^ 250 bp paired-end reads for each sample with high mean quality Phred scores ranging from 35 to 37 (Table 1). Ligation and the 1 ng PE RNA titration sample returned less paired-end reads, 1.3 and 1.9 million reads, respectively (Table 1). For the 1 ng sample this was most likely due to the limited amount of template and generated repertoire library (Fig. S1E in File S1, the entire library was sequenced while for all other samples calculated amounts allowing equal read return were loaded on the flow-cell). Since the ligation-derived library contained only partially adapter-ligated product, it was more difficult to load exact amounts of sequencing-ready product, which was likely responsible for the lower read return.

Paired-end reads were pre-processed by overlap assembly (pairing rates of raw 2×250 bp reads reached an average of ≈97%, Table 1) and submitted to the open-access IMGT/HighV-QUEST software platform [31], [32] in order to obtain proper VDJ and CDR3 annotation. We primarily focused on using CDR3 exact (100% identity) amino acid sequences as clonal identifiers. CDR3s could be mapped to ≈93% of pre-processed reads demonstrating the high quality of the HTS datasets (Table 1). As a final filtering step, singleton CDR3s and CDR3s shorter than 4 amino acids were removed from each dataset to reduce the impact of PCR and sequencing errors on downstream statistical analyses [37].

### Investigated Adapter Addition Methods Yielded Comparable HTS Datasets

**Figure 3:**
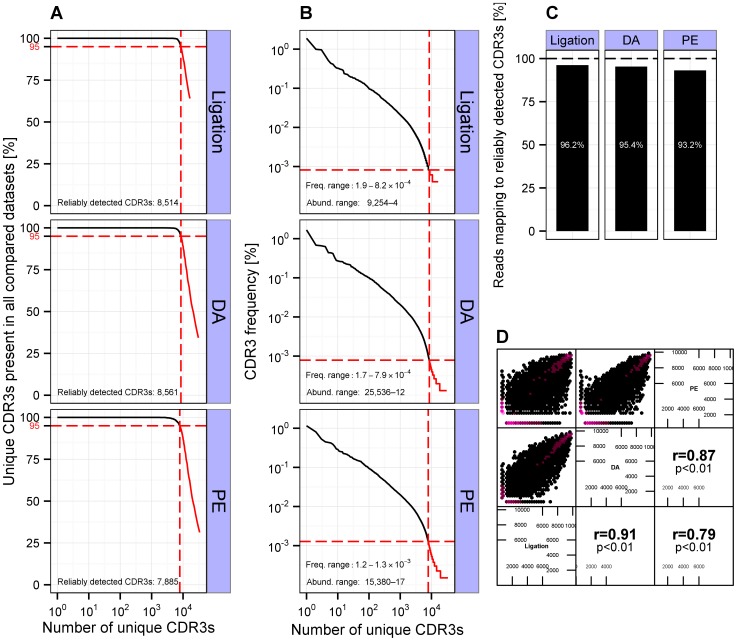
Ligation-, DA-, and PE-based adapter addition methods yield highly comparable HTS datasets. (A) Reliably detected CDR3s were determined as described in Fig. 2 using a 95% CDR3 reliable detection cut-off. On average, ≈8,300 CDR3s were reliably detected with the three different methods. The red dashed line marks the 95% detection cut-off separating reliably detected clones (black line) from non-reliably detected ones (red line). (B) Average frequency and abundance ranges (≈1.6–5.8×10^−4^% and ≈17,000–11 reads per unique CDR3, respectively) of reliably detected CDR3s. (C) Reliably detected CDR3s corresponded to an average of ≈95% of total sequencing reads for the three different methods. (D) For each method, ranks of reliably detected CDR3s were determined and displayed by assigning the highest rank to the CDR3 with the highest abundance; analogous to the hypothetical dataset presented in Fig. 2 the highest frequency CDR3 would be assigned the rank 10, the second highest CDR3 the rank 9 and so forth. Pairwise Spearman’s rank correlation coefficients for Ligation, DA, and PE were high (r≥0.79). To circumvent overplotting, correlation plots are displayed using hexagons–purple indicates where data points accumulate.

We compared the three different methods of sequencing adapter addition (Ligation/DA/PE) by means of a previously established method for the reliable determination of antibody clones, which is based on the simultaneous existence of clones (CDR3s) in each of the investigated datasets [27] (Fig. 2B) and benchmarked those results to a technical replicate. Briefly, for each clone presence or absence in the other datasets was recorded while moving down a list of frequency-ranked CDR3s (IMGT output, Fig. 2). Those clones that belonged to the highest frequency set of clones with 95% of the contained clones present in the other datasets were regarded as “reliably detected” and compiled into a list (Fig. 2B). After application of a 95% cut-off to the datasets obtained with ligation, DA, and PE methods, an average of ≈8,300 CDR3 clones were reliably detected per method (Fig. 3A). These CDR3s spanned over an average frequency range of ≈1.6–5.8×10^−4^% and a corresponding average abundance range of ≈17,000–11 reads per unique CDR3 (Fig. 3B). These ranges of reliable detection imply that the CDR3s that lay below the 95%-threshold, and were thus not reliably detected across datasets, were almost 4 orders of magnitude less abundant than the highest frequency clones. Indeed, the reliably detected clones mapped to over 93% of total reads (Fig. 3C) demonstrating the high overlap of the three HTS datasets as well as an enormous depth of reliable detection. In a final step, we established that in addition to a highly similar clonal composition, the clonal ranking was also conserved across methods; the Spearman rank correlation coefficient ranged between r = 0.93–0.79 (Fig. 3D). While the number of reliably detected CDR3s for the three methods (Ligation/DA/PE) constituted 70% of those of a technical duplicate (≈12,400 CDR3s detected in the DA-duplicate, Fig. S2A in File S1), ranges of reliable detection stretched in both cases over almost 4 orders of magnitude, with CDR3s mapping to similar amounts of total reads (≈97% of total reads, Fig. S2C in File S1). Pairwise comparisons indicated that the ligation sample lowered the amount of reliably detectable CDR3s since the DA–PE comparison was in the range of the technical duplicate for both CDR3 composition and ranking (Fig. S3–S5 in File S1). Of interest, variations in the PE protocol did not affect HTS datasets (Fig. S6 in File S1); samples PE1–3 showed similar statistics (detected CDR3s, range of detection, corresponding reads, and rank correlations) to the technical duplicate (Fig. S2 in File S1). To summarize, the HTS analysis demonstrated high comparability of the three methods of adapter addition. Thus, in contrast to experimental considerations, differences between adapter addition methods on the HTS level were found to be negligible.

Furthermore, in order to assess the influence of degenerate primers on VDJ sequence diversity, the full-length amino acid sequences of both complete antibody variable regions (VDJ) as well as primer-trimmed VDJs were used as clonal identifiers and reliable detection analyses were performed. We found differences between complete and primer-trimmed VDJs in average numbers of reliably detected VDJs (non-trimmed: ≈35,400, trimmed: ≈13,500; Fig. S8A/E in File S1), frequency ranges of reliable detection (Fig. S8B/C/F/G in File S1) and corresponding rank correlations (non-trimmed: r = 0.68, trimmed: r = 0.9, Fig. S8D/H in File S1). The introduced increase in VDJ diversity as well as decrease in VDJ rank correlation was likely the result of primer mis-annealing during PCR. The loss of information on the full VDJ sequence due to primer trimming and mis-annealing may be avoided by using VDJ amplification approaches that do not rely on primers annealing in the FR1 region [20], [40]–[42].

### Minimal Amounts of Total RNA Input Preserved the Antibody Repertoire Composition

**Figure 4:**
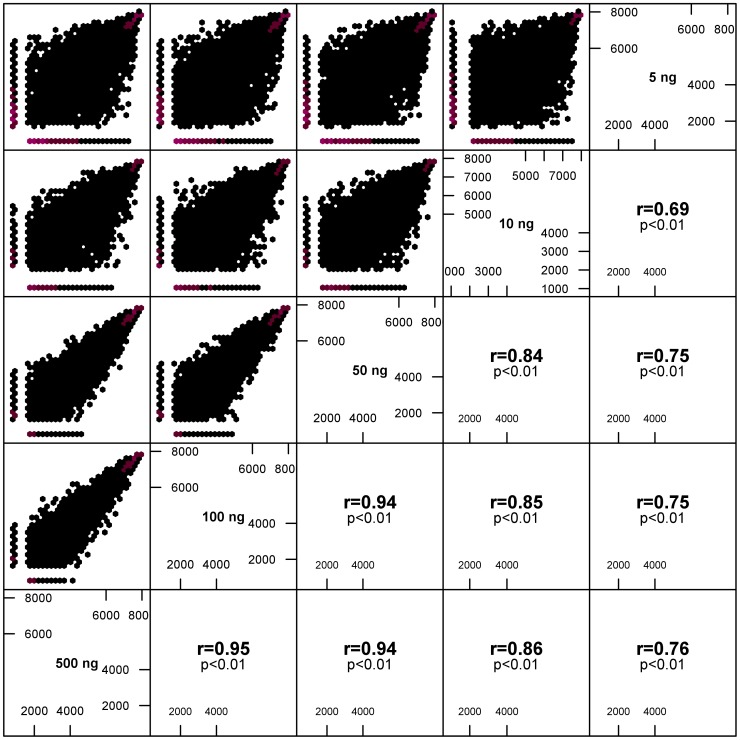
Comparison of HTS datasets from the RNA titration experiment (500–5 ng) using the PE method. Spearman’s rank correlation of reliably detected CDR3s decreased with decreasing RNA input (r = 0.95–0.69). Antibody amplicon libraries prepared using PE and differing amounts of RNA input (500–5 ng) were sequenced (see *Methods*). CDR3 ranks were determined as detailed in Fig. 3. Average numbers of reliably detected CDR3s, frequency and abundance ranges, and the percentage of reads corresponding to reliably detected CDR3s are summarized in Table S1 in File S1.

As established above (Table 1), PE enabled HTS with ultra-low RNA input (amounts as little as 1 ng of total RNA produced a gel-extractable library). We performed HTS on all titrated samples and investigated the effect of lowering the amount of input RNA on the resulting HTS datasets. Sequencing the libraries generated from the RNA titration revealed ≈6,200 reliably detected CDR3s in each titrated sample from 500–5 ng (Table S1 in File S1). This resulted in average frequency and abundance ranges of reliable detection of ≈1.1–2.5×10^−3^% and ≈15,000–33 reads per unique CDR, respectively, extending across 3 orders of magnitude (Table S1 in File S1). Reliably detected CDR3s mapped to ≈91% of sequencing reads (Table S1 in File S1). Rank correlation coefficients among 500–5 ng samples ranged between r = 0.95–0.69 (Fig. 4); however, they decreased as a function of decreasing total RNA input. Including the 1 ng dataset in the reliable detection analysis (500–1 ng) dramatically lowered the average of reliably detected clones from ≈6,200 to ≈1,000 (Fig. S7A in File S1). Additionally, the 1 ng RNA input showed low rank correlation coefficients (r = 0.41–0.38; Fig. S7D in File S1) when compared to all other titration samples. Although including 1 ng in the data analysis led to a lower number of reliably detected CDR3 clones and low rank correlations, the 30 highest frequency CDR3s of 1 ng were highly consistent with the 500–5 ng input libraries regarding both composition (21 out of top 30 clones were found in all 6 datasets, Table S2 in File S1) and ranking (mean r = 0.81).

In summary, decreasing total RNA inputs from 500–5 ng preserved the top CDR3s over several orders of magnitude showing differences in CDR3 repertoires below rank ≈6,200. CDR3 rank correlation coefficients for 500–5 ng are high but deteriorate with decreasing RNA input. However, reducing the RNA input to 1 ng disrupted the clonal overlap (number of reliably detected CDR3s) and CDR3 ranking (Fig. S7 in File S1). Nevertheless, the 30 highest frequency clones maintained consistent composition and ranking across the entire range of RNA input (500–1 ng).

## Discussion

In this work, we presented a comprehensive evaluation of two PCR (DA, PE)- and one ligation-based method for the addition of Illumina sequencing adapters to antibody variable gene libraries examining the effects with respect to yield, practicality, and effect on resulting HTS datasets. We compared HTS datasets of antibody repertoire libraries generated with the three methods of adapter addition and determined the extent of CDR3 repertoire similarity by leveraging the concept of reliable detection [27]. We found that on average ≈8,300 CDR3s were reliably detected across the methods mapping to >93% of pre-processed sequencing reads (Fig. 3). Pairwise comparisons between HTS datasets (DA/PE (Fig. S3 in File S1), DA/Ligation (Fig. S4 in File S1), PE/Ligation (Fig. S5 in File S1)) suggested that ligation was lowering the CDR3 overlap, which was likely due to lower read numbers compared to DA and PE (Table 1). Indeed, DA and PE showed an average number of reliably detected CDR3s (≈11,500, Fig. S3 in File S1) close to that of the DA technical duplicate (≈12,200, Fig. 2). Collectively, the reliably detected CDR3s in HTS datasets generated using ligation, DA, and PE defined a range of reliability stretching over nearly 4 orders of magnitude (≈1–10^−4^%) within which CDR3 ranking was highly conserved (r≥0.79, Fig. 3D). This reliability range extended over as many orders of magnitude as that of the technical duplicate (Fig. 2) showing that ligation-, DA-, and PE-HTS datasets were highly comparable and generated statistically equivalent repertoires. Importantly, we were able to establish the comparability of methods despite differing sequencing depths (Table 1) by employing both the CDR3 overlap and the range of reliable detection as criteria for methodological equivalence (e.g. Fig. 3 and Table S1 in File S1).

The finding that all three methods of adapter addition resulted in comparable and statistically equivalent HTS datasets allowed for their evaluation based on experimental performances: yield (amount of final library), efficiency (yield related to RNA input and PCR cycle number), and overall practicality. Of the methods tested, ligation required the highest amount of starting RNA material due to the fact that a minimum input of 1 µg of preamplified DNA library was necessary as input for the ligation reaction (manufacturer’s recommendation). Despite the high amount of input RNA and the high number of PCR cycles, the final extracted product yield was low (≈20 ng, Table 1) and did not consist of a completely adapter-ligated, HTS-ready library. Combining all experimental steps, ligation required nearly twice as much preparation time as the DA method (6–7 h starting with cDNA) but was a straightforward protocol that did not require any specific primer design and purchase. While the kit price itself was affordable, the pronounced effort necessary to generate enough DNA library in addition to the recurrent costs for the kit made this method less practical and potentially more costly in the long term for frequent HTS runs. Moreover, switching from Taq to a polymerase that does not add 3′A-overhangs to the DNA product will require additional modifications of the library (A-tailing), which may ultimately have a negative impact on yield and efficiency. Another drawback is the unknown ratio of adapter-ligated to non-ligated product in the final extracted library, thus impeding read-equilibrated HTS runs. It should be noted that kits are now available that offer the ligation addition of Illumina adapters with starting DNA inputs as little as 5 ng (NEBNext Ultra, NEB). However, in terms of RNA input our optimized PE protocol, demonstrated to work with 5–1 ng RNA, would still outperform the new kits. In terms of preparation time and practicality these ligation kits are comparable to the kit used in this study.

The DA method readily allowed for library preparations with a more standard input of 500 ng total RNA (Table 1) [16], [37]–[39] yielding a library that did not require any further treatment, thereby proving to be a fast (3 h total preparation time), single-step method that could be reliably used with moderate amounts of input RNA (Table 1). Furthermore, the near-complete incorporation of sequencing adapters not only increased the overall efficiency of this method, but also allowed for a direct calculation of sequencing-ready DNA molecules enabling read-equilibrated multiplexed HTS runs. For frequent use, DA might become a low-priced method, since the possibility to generate multiple libraries could amortize in the long run the higher initial costs of the longer primer mixes.

The PE method was designed similarly to Wang and colleagues [26] enabling a decrease in primer length of the forward mix (PCR1) from 90 to 40 bases compared to DA (Fig. 1, Table S3 in File S1). Initial amplification was performed with a reduced number of PCR cycles, while uniformly high amplification was achieved in PCR2 by using only two primers that incorporated universal and index Illumina adapter sequences and annealed to the GC-rich overhang introduced during PCR1. We found that protocol variations (shifting cycle numbers between PCRs and reducing overall numbers of PCR cycles) did not affect the resulting HTS datasets (Fig. S6 in File S1). However, as expected, a reduction in cycle numbers consequentially resulted in lower library yields (Table 1). The high efficiency of the PE method was demonstrated by the fact that a 25-fold higher library yield was obtained from the same amount of starting RNA (500 ng) using the same number of total PCR cycles compared to DA (PE1/3; Table 1). Even more so, the PE method was capable of generating a higher library yield than the ligation method while requiring less preparation time. Therefore, our optimized protocol for PE may be the method of choice in case of limited amounts of RNA or very small cell populations (e.g., rare B-cell populations from humans or mice) [43], [44].

RNA titration experiments were performed to probe the impact of RNA input on HTS datasets. We titrated total RNA inputs over 2 orders of magnitude (500–1 ng, Table 1) from the standard total RNA input of 500 ng [16], [37]–[39]. Following PE-based library preparation and HTS, we found that for 500–5 ng the average frequency range of reliable detection extended over 3 orders of magnitude (≈1–10^−3^%) with a diverging CDR3 repertoire composition below a CDR3 frequency of ≈2.5×10^−3^% and abundance of 32 reads per unique CDR3 (Table S1 in File S1). CDR3 rank correlations for 500–5 ng ranged between r = 0.95–0.69 and decreased as a function of decreasing total RNA input (Fig. 4). These results suggest that only the higher frequency CDR3s were recovered with high confidence when sequencing very low RNA samples (Table S2 in File S1). Indeed, although the 1 ng sample delivered less reads and demonstrated a low correlation with 500–5 ng, it still returned a very similar set of 30 highest frequency CDR3s when compared to those found in the 500–5 ng samples (mean r = 0.81, Table S2 in File S1), thus suggesting it is as a suitable method for studies where RNA is limited and only the top clones are of interest (e.g., monoclonal antibody discovery) [23], [45].

In summary, this report provides significant details on methods for the quantitative analysis of humoral immune responses by HTS of antibody variable genes. As of yet, many studies performing antibody HTS have relied on non-FACS sorted cells (e.g., human PBMCs or total splenocytes), where consequentially RNA amounts were rarely limited [37], [46], [47]. However, answering more detailed questions about immunological repertoires, regarding diversity and development, monoclonal antibody discovery, and vaccination, will require the HTS of small or even rare T/B-cell sub-populations in bulk [43], [44], [48]. Since immune cell populations of interest can be very small (especially in mice) [48]–[51] and RNA content therefore limited, highly efficient amplification methods such as our optimized PE method can pave the way towards systems immunology studies that exploit HTS for the micro-dissection of immune repertoires and responses.

Furthermore, this report is highly relevant for amplicon HTS regarding experimental library preparation and statistical interpretation of HTS datasets. We described two PCR-based methods of adapter addition, both of which exhibited strong experimental advantages compared to the ligation method, thereby predestining them as future standard of adapter addition. On the level of HTS, employing the framework of reliable detection, we concluded that all three examined methods yielded comparable and nearly equivalent datasets, thus underlining the advantages of the PCR-based methods of adapter addition. The framework of reliable clonal detection did not only enable the quantitation of absolute numbers of reliably shared CDR3s across HTS datasets but also gave rise to ranges of reliable detection. Specifically, these ranges implicated the determination of minimal clonal frequencies, below which HTS datasets of tested methods and conditions showed a divergence in clonal composition. We believe that these strict, objective, and unbiased reliability thresholds will become standard and essential components of future comparative HTS analyses.

## Supporting Information

File S1 **File S1 includes the following: Figure S1.** Amplicon libraries on 1% agarose gel. **Figure S2.** DA duplicates (DA1/DA2, technical replicates) yield highly comparable HTS datasets. **Figure S3.** Pairwise comparison of HTS datasets from antibody repertoire libraries prepared using the DA and PE method. **Figure S4.** Pairwise comparison of HTS datasets from antibody repertoire libraries prepared using the ligation and DA method. **Figure S5.** Pairwise comparison of HTS datasets from antibody repertoire libraries using the ligation and PE method. **Figure S6.** Variations in the PE protocol have minimal effects on HTS datasets. **Figure S7.** Comparison of HTS datasets from antibody repertoire libraries prepared using the PE method and lowering the amounts of total RNA input (500–1 ng). **Figure S8.** VDJ primer trimming reduces VDJ diversity. **Table S1.** RNA titration (500–5 ng) using the PE method. **Table S2.** The 30 highest ranked CDR3 amino acid sequences of all RNA titration datasets are shown in a color-coded manner with respective frequencies. **Table S3.** List of all primers used for ligation, DA, and PE.
(DOCX)
