# Supplementary material for: Comprehensive Evaluation and Optimization of Amplicon Library Preparation Methods for High-Throughput Antibody Sequencing
Source: PLoS One. 2014 May 8;9(5):e96727. doi: 10.1371/journal.pone.0096727 (PMC4014543; doi:10.1371/journal.pone.0096727)
Supplement: File S1 — includes the following: Figure S1. Amplicon libraries on 1% agarose gel. Figure S2. DA duplicates (DA1/DA2, technical replicates) yield highly comparable HTS datasets. Figure S3. Pairwise comparison of HTS datasets from antibody repertoire libraries prepared using the DA and PE method. Figure S4. Pairwise comparison of HTS datasets from antibody repertoire libraries prepared using the ligation and DA method. Figure S5. Pairwise comparison of HTS datasets from antibody repertoire libraries using the ligation and PE method. Figure S6. Variations in the PE protocol have minimal effects on HTS datasets. Figure S7. Comparison of HTS datasets from antibody repertoire libraries prepared using the PE method and lowering the amounts of total RNA input (500–1 ng). Figure S8. VDJ primer trimming reduces VDJ diversity. Table S1. RNA titration (500–5 ng) using the PE method. Table S2. The 30 highest ranked CDR3 amino acid sequences of all RNA titration datasets are shown in a color-coded manner with respective frequencies. Table S3. List of all primers used for ligation, DA, and PE. (DOCX) [file pone.0096727.s001.docx]

# Supporting Information


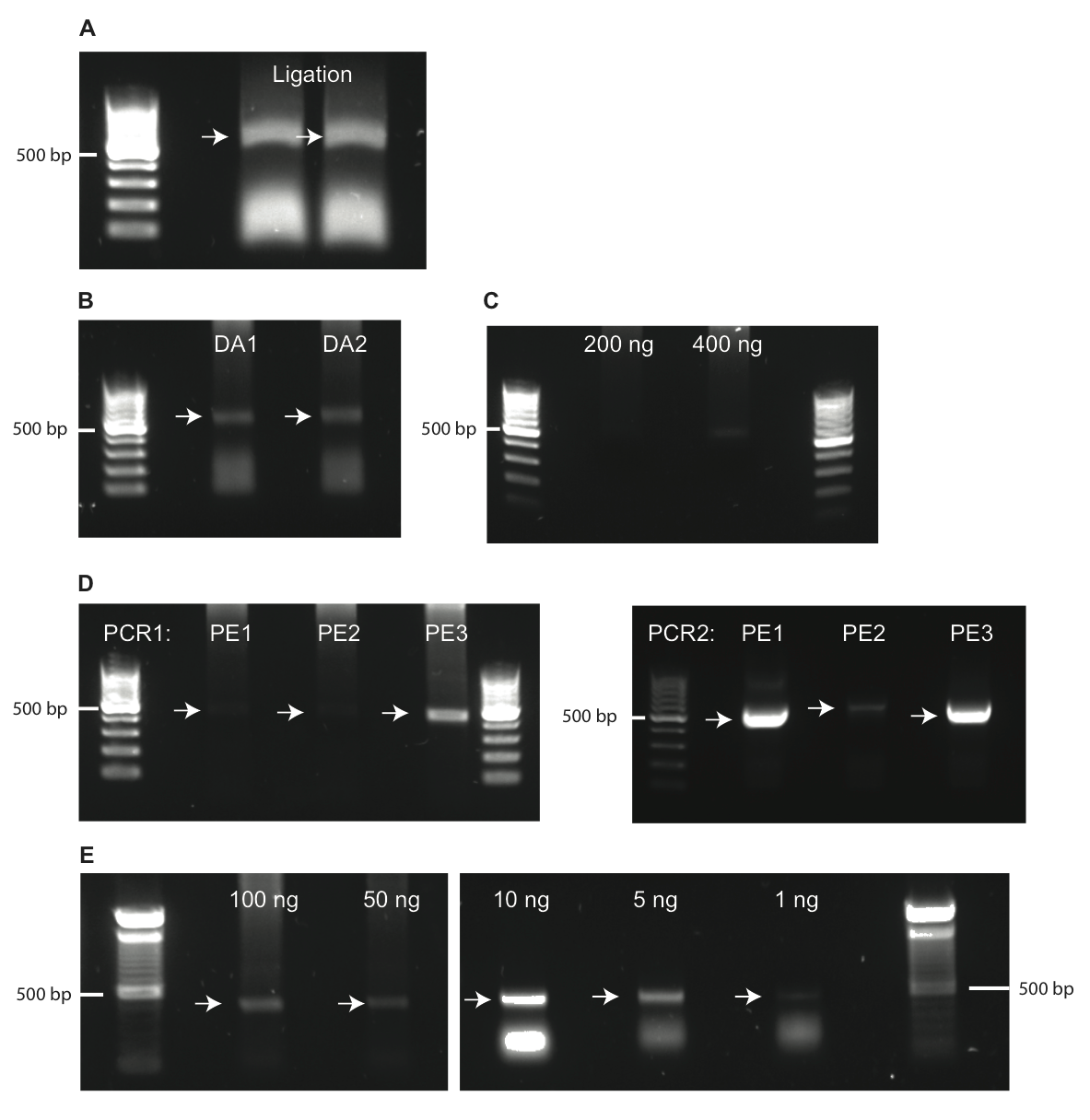


Figure S 1. Amplicon libraries on 1% agarose gel. (A) Final product of ligation method. Both indicated bands were purified. (B) Duplicate libraries (DA1/DA2) generated using the DA method. (C) DA titration with 200 and 400 ng. (D) PE products after PCR1 and PCR2. (E) RNA titration using standard PE3 PCR protocol for high amount RNA samples (100 ng and 50 ng) and an extended protocol for low amount RNA samples (10–1 ng, see *Methods*). Bands are shown after PCR2. Bands between 500–600 bp in length (indicated by white arrows) were excised and gel-purified.

Figure S 2. DA duplicates (DA1/DA2, technical replicates) yield highly comparable HTS datasets. (A) Reliably detected CDR3s were determined as described in Fig. 2 using a 95% reliable detection cut-off. On average, ≈12,200 CDR3s were reliably detected. (B) Their corresponding average frequency and abundance ranges are shown (≈1.7–3.3x10^-4^% and ≈24,000–5 reads per unique CDR3, respectively). (C) Reliably detected CDR3s corresponded to ≈97% of total sequencing reads. (D) The Spearman rank correlation coefficient was high (r=0.93).

Figure S 3. Pairwise comparison of HTS datasets from antibody repertoire libraries prepared using the DA and PE method. (A) Reliably detected CDR3s were determined as described in Fig. 2 using a 95% reliable detection cut-off. On average, ≈11,500 CDR3s were reliably detected. (B) Their corresponding average frequency and abundance ranges are shown (≈1.5–3.9x10^-4^ % and ≈20,000–5 reads per unique CDR3, respectively). (C) Reliably detected CDR3s corresponded to ≈96% of total sequencing reads. (D) The Spearman rank correlation coefficient was high (r=0.90).

Figure S 4. Pairwise comparison of HTS datasets from antibody repertoire libraries prepared using the ligation and DA method. (A) Reliably detected CDR3s were determined as described in Fig. 2 using a 95% reliable detection cut-off. On average, ≈8,700 CDR3s were reliably detected. (B) Their corresponding average frequency and abundance ranges are shown (≈1.8–8.1x10^-4^% and ≈17,000–8 reads per unique CDR3, respectively). (C) Reliably detected CDR3s corresponded to ≈96% of total sequencing reads. (D) The Spearman rank correlation coefficient was high (r=0.86).

Figure S 5. Pairwise comparison of HTS datasets from antibody repertoire libraries using the ligation and PE method. A) Reliably detected CDR3s were determined as described in Fig. 2 using a 95% CDR3 reliable detection cut-off. On average, ≈8,700 CDR3s were reliably detected. (B) Their corresponding average frequency and abundance ranges are shown (≈1.6–3.7x10^-4^% and ≈12,500–10 per unique CDR3, respectively). (C) Reliably detected CDR3s corresponded to ≈95% of total sequencing reads. (D) The Spearman rank correlation coefficient was high (r=0.79).

Figure S 6. Variations in the PE protocol have minimal effects on HTS datasets. PE1 and PE2 were prepared with 8 cycles in the last annealing temperature step of PCR1 (16 total cycles) and either amplified with 12 (PE1) or 8 (PE2) total cycles in PCR2, resulting in an overall number of 28 and 24 cycles, respectively. For PE3, 4 cycles were switched from PCR2 to PCR1, having 12 cycles in the last annealing temperature step of PCR1 (20 total cycles) and a total of 8 cycles in PCR2, resulting in an overall cycle number of 28 (equal cycle number to PE1 and standard conditions used for ligation and DA). (A) Reliably detected CDR3s were determined as described in Fig. 2 using a 95% CDR3 reliable detection cut-off. On average, ≈13,000 CDR3s were reliably detected. (B) Their corresponding average frequency and abundance ranges are shown (≈1.1–2.7x10^-4^% and ≈20,000–5 per unique CDR3, respectively). (C) Reliably detected CDR3s corresponded to ≈96% of sequencing reads. (D) The Spearman rank correlation coefficients were high (r≈0.93).

Figure S 7. Comparison of HTS datasets from antibody repertoire libraries prepared using the PE method and lowering the amounts of total RNA input (500–1 ng). Including the 1 ng dataset into the comparison of 500–5 ng datasets reduced the number of reliably detected CDR3s and CDR3 rank correlation (Fig. 4, Table S1). (A) Reliably detected CDR3s were determined as described in Fig. 2 using a 95% reliable detection cut-off. On average, ≈1,000 CDR3s were reliably detected. (B) Their corresponding average frequency and abundance ranges are shown (≈1.1–2.3x10^-2^% and ≈14,000–300 per unique CDR3, respectively). (C) Reliably detected CDR3s corresponded to ≈52% of total sequencing reads. (D) The Spearman rank correlation coefficients ranged between r=0.92 and r=0.38.

Figure S 8. VDJ primer trimming reduces VDJ diversity. The effect of primer trimming on VDJ diversity was assessed using the DA method duplicate (DA1/DA2). Primer trimming of VDJ regions was performed as detailed in *Methods*. (A/E) Reliable detected VDJs were determined as described in Fig. 2 using a 95% VDJ reliable detection cut-off. On average, ≈35,400 and ≈13,500 VDJs were reliably detected for non-trimmed and trimmed HTS datasets, respectively. (B/F) Their corresponding average frequency and abundance ranges are shown. (C/G) Reliably detected VDJs corresponded to ≈80/≈88% of total sequencing reads. (D/H) The Spearman’s rank correlation coefficients were r=0.68 and r=0.90 for non-trimmed and trimmed HTS datasets, respectively.

Table S 1. RNA titration (500–5 ng) using the PE method. On average, ≈6,200 CDR3s were reliably detected, which extended over average frequency and abundance ranges of ≈1.1–2.5x10^-3^% and ≈15,000–34 reads per unique CDR3, respectively. Reliably detected CDR3s corresponded to ≈92% of total sequencing reads. The corresponding Spearman rank correlation coefficients are shown in Fig. 4.

| HTS dataset  (PE) | Number of reliably detected CDR3s | Frequency  range [%] | Abundance range | Reads mapping to reliably detected CDR3s [%] |
| --- | --- | --- | --- | --- |
| 500 ng | 6,363 | 1.2–2.4x10^-3^ | 15,380–32 | 93.4 |
| 100 ng | 6,327 | 1.1–2.5x10^-3^ | 13,000–30 | 91.0 |
| 50 ng | 6,333 | 1.2–2.5x10^-3^ | 10,771–23 | 92.4 |
| 10 ng | 5,858 | 1.1–2.7x10^-3^ | 17,175–44 | 92.1 |
| 5 ng | 6,155 | 1.0–2.2x10^-3^ | 18,636–40 | 90.5 |
| Average | 6,207 | 1.1–2.5x10^-3^ | 14,992–34 | 91.9 |

Table S 2. The 30 highest ranked CDR3 amino acid sequences of all RNA titration datasets are shown in a color-coded manner with respective frequencies. Color code based on CDR3s from the 500 ng sample: Top 1­–5: bright fill, white font; Top 6–10: bright fill, black font; Top 11–15: Dark fill, white font; Top 16–20: grey fill, white font; Top 21–25: pastel fill, black font; Top 26–30: yellow fill, black font. Clones present in 5 out of 6 datasets are marked in red font. Clones, present in less than five out of the six datasets are not color-indicated. The mean Spearman rank correlation coefficient of datasets 500–1ng is r=0.81. Freq.: frequency of clones in percent.

|  | **500 ng** | | **100 ng** | | | **50 ng** | | | **10 ng** | | **5 ng** | | **1ng** | |
| --- | --- | --- | --- | --- | --- | --- | --- | --- | --- | --- | --- | --- | --- | --- |
|  | Sequence | Freq. | Sequence | Freq. | Sequence | | Freq. | Sequence | | Freq. | Sequence | Freq. | Sequence | Freq. |
| 1 | ARWEYYAMDY | 1.156 | ARWEYYAMDY | 1.070 | ARWEYYAMDY | | 1.153 | ARWEYYAMDY | | 1.055 | ARWEYYAMDY | 1.021 | ARWEYYAMDY | 1.187 |
| 2 | ARLNYYGNLFV | 0.802 | ARLNYYGNLFV | 0.890 | ARLNYYGNLFV | | 0.927 | ARLNYYGNLFV | | 0.811 | ARLNYYGNLFV | 0.916 | ARLNYYGNLFV | 0.776 |
| 3 | ARHAYYDQTEVSFVY | 0.528 | ARHAYYDQTEVSFVY | 0.630 | ARHAYYDQTEVSFVY | | 0.594 | ARHAYYDQTEVSFVY | | 0.576 | ARHAYYDQTEVSFVY | 0.635 | ARHAYYDQTEVSFVY | 0.739 |
| 4 | ARLDLFAY | 0.449 | ARHHRYAYYFDY | 0.474 | ARDVGYGNYFVY | | 0.467 | ARHHRYAYYFDY | | 0.518 | ARHHRYAYYFDY | 0.497 | ARHHRYAYYFDY | 0.565 |
| 5 | ARHHRYAYYFDY | 0.433 | ARDVGYGNYFVY | 0.443 | ARLDLFAY | | 0.463 | ARLDLFAY | | 0.449 | ARDVGYGNYFVY | 0.415 | ASYRYGWYFDV | 0.532 |
| 6 | ASYRYGWYFDV | 0.420 | ASYRYGWYFDV | 0.410 | ARHHRYAYYFDY | | 0.457 | ARDVGYGNYFVY | | 0.426 | ARLDLFAY | 0.414 | ARDVGYGNYFVY | 0.440 |
| 7 | ARDVGYGNYFVY | 0.388 | ARLDLFAY | 0.403 | ASYRYGWYFDV | | 0.374 | ASYRYGWYFDV | | 0.354 | ASYRYGWYFDV | 0.391 | ATYDGYYFDY | 0.392 |
| 8 | ARSGKVRNAMDY | 0.342 | ARSGKVRNAMDY | 0.341 | ARSGKVRNAMDY | | 0.348 | ARSGKVRNAMDY | | 0.314 | ARSGKVRNAMDY | 0.319 | AAYYYGSSYGFAY | 0.338 |
| 9 | ARYRLGTYFDY | 0.296 | ATYDGYYFDY | 0.288 | ATYDGYYFDY | | 0.332 | ARLIYGSSYIVDF | | 0.276 | ARWELGRAWFAY | 0.277 | ATYDGYCFDY | 0.316 |
| 10 | ASYVYGYYFDY | 0.288 | ARYRLGTYFDY | 0.286 | ARYRLGTYFDY | | 0.311 | ATYDGYYFDY | | 0.276 | ARSGSSSGYFDY | 0.262 | ASYVYGYYFDY | 0.311 |
| 11 | TREGDSPFAY | 0.261 | ASYVYGYYFDY | 0.268 | ASYVYGYYFDY | | 0.289 | ASYVYGYYFDY | | 0.263 | ATYDGYYFDY | 0.254 | ARGGISVDY | 0.302 |
| 12 | ATYDGYYFDY | 0.254 | ARLIYGSSYIVDF | 0.253 | ARGGISVDY | | 0.273 | ARGGISVDY | | 0.263 | ARDINYDIYWYFDV | 0.250 | ARYRLGTYFDY | 0.287 |
| 13 | ARLIYGSSYIVDF | 0.251 | ARLGYYGWFAY | 0.252 | ARLGYYGWFAY | | 0.267 | ARLGYYGWFAY | | 0.262 | ARGGISVDY | 0.248 | ARDINYDIYWYFDV | 0.279 |
| 14 | ARGGISVDY | 0.250 | ARGGISVDY | 0.249 | ARLGYYGYFAY | | 0.262 | ARYRLGTYFDY | | 0.262 | ASYVYGYYFDY | 0.239 | ANYYYGSSLFAY | 0.278 |
| 15 | TTGFAY | 0.226 | ARSGSSSGYFDY | 0.249 | ARSGSSSGYFDY | | 0.258 | TTGFAY | | 0.253 | ARYRLGTYFDY | 0.235 | ARLDLFAY | 0.273 |
| 16 | AAYYYGSSYGFAY | 0.225 | ARLGYYGYFAY | 0.249 | TTGFAY | | 0.250 | AREARTTARFAY | | 0.252 | ARLGYYGGFAY | 0.235 | ARLIYGSSYIVDF | 0.268 |
| 17 | ARLGYYGWFAY | 0.222 | AREARTTARFAY | 0.242 | ARLGYYGGFAY | | 0.246 | ATYDGYCFDY | | 0.252 | ARLGYYGYFAY | 0.232 | ARLGYYGYFAY | 0.253 |
| 18 | ARSGSSSGYFDY | 0.220 | ARLGYYGGFAY | 0.238 | ARLIYGSSYIVDF | | 0.241 | ARLGYYGYFAY | | 0.244 | TRGGNWEDFDY | 0.222 | ARLGYYGGFAY | 0.250 |
| 19 | AREARTTARFAY | 0.215 | TREGDSPFAY | 0.231 | ATYDGYCFDY | | 0.229 | ARSGSSSGYFDY | | 0.242 | ARLGYYGWFAY | 0.219 | ARDKGRVFFDY | 0.239 |
| 20 | ARLGYYGYFAY | 0.215 | ARDKGRVFFDY | 0.226 | AREARTTARFAY | | 0.228 | TRGGNWEDFDY | | 0.231 | TREGDSPFAY | 0.217 | TTGFAY | 0.236 |
| 21 | ARLGYYGGFAY | 0.207 | ATYDGYCFDY | 0.224 | TREGDSPFAY | | 0.228 | AAYYYGSSYGFAY | | 0.213 | ATYDGYCFDY | 0.215 | ARLGYYGWFAY | 0.228 |
| 22 | ARDKGRVFFDY | 0.203 | TTGFAY | 0.217 | ARDKGRVFFDY | | 0.218 | ARLGYYGGFAY | | 0.209 | ARDYGSSHFDH | 0.209 | ARSGKVRNAMDY | 0.224 |
| 23 | ATYDGYCFDY | 0.200 | AAYYYGSSYGFAY | 0.215 | ARLDYYGYLNY | | 0.211 | ARWELGRAWFAY | | 0.202 | TTGFAY | 0.205 | ARGGANVDY | 0.222 |
| 24 | ARWELGRAWFAY | 0.193 | TRGGNWEDFDY | 0.212 | AAYYYGSSYGFAY | | 0.209 | TREGDSPFAY | | 0.202 | ASYRYAWYFDV | 0.204 | AREARTTARFAY | 0.219 |
| 25 | ARDYGSSHFDH | 0.186 | ARFPMDY | 0.194 | ARFPMDY | | 0.207 | ARDSFLITRALDY | | 0.200 | ARLIYGSSYIVDF | 0.204 | ARKGAMITHYYAMDY | 0.217 |
| 26 | ARSGGYGNYLAWFAY | 0.183 | SRHYYSTPYYAMDY | 0.192 | TRGGNWEDFDY | | 0.198 | ARDKGRVFFDY | | 0.191 | ARFPMDY | 0.202 | TIGGFDY | 0.215 |
| 27 | ARFPMDY | 0.182 | ARDSFLITRALDY | 0.190 | ARWELGRAWFAY | | 0.192 | ARLDYYGYLNY | | 0.191 | AREARTTARFAY | 0.201 | TREGTYDGCSGHFDY | 0.214 |
| 28 | ARGGHDFSLDY | 0.182 | ARGGHDFSLDY | 0.189 | ARLSYYGRFAY | | 0.191 | ARFPMDY | | 0.188 | ARSGGYGNYLAWFAY | 0.201 | ARGGHDFSLDY | 0.214 |
| 29 | SRHYYSTPYYAMDY | 0.179 | ARWELGRAWFAY | 0.187 | AKNYYYGYVAY | | 0.185 | AKNYYYGYVAY | | 0.183 | ARDKGRVFFDY | 0.198 | ARWELGRAWFAY | 0.197 |
| 30 | ARDSFLITRALDY | 0.171 | ARSGGYGNYLAWFAY | 0.183 | ARQGYYGKGDY | | 0.185 | SRHYYSTPYYAMDY | | 0.182 | ARLDYYGYLNY | 0.195 | ARHGITRLLDY | 0.196 |

Table S 3. List of all primers used for ligation, DA, and PE. fw: forward, rv: reverse, UAd: universal adapter, Idx: index, RC: reverse complement.

**Ligation** ^1^

| **DA** |  |
| --- | --- |
|  |  |
| **IgH Universal Forward** | **TruSeq Universal Adapter + Diversity region + VH 5' specific region** |
|  |  |
| IgH-UAd-fw1 | Universal Adapter - NNNN GAKGTRMAGCTTCAGGAGTC |
| IgH-UAd-fw2 | Universal Adapter - NNNN GAGGTBCAGCTBCAGCAGTC |
| IgH-UAd-fw3 | Universal Adapter - NNNN CAGGTGCAGCTGAAGSASTC |
| IgH-UAd-fw4 | Universal Adapter - NNNN GAGGTCCARCTGCAACARTC |
| IgH-UAd-fw5 | Universal Adapter - NNNN CAGGTYCAGCTBCAGCARTC |
| IgH-UAd-fw6 | Universal Adapter - NNNN CAGGTYCARCTGCAGCAGTC |
| IgH-UAd-fw7 | Universal Adapter - NNNN CAGGTCCACGTGAAGCAGTC |
| IgH-UAd-fw8 | Universal Adapter - NNNN GAGGTGAASSTGGTGGAATC |
| IgH-UAd-fw9 | Universal Adapter - NNNN GAVGTGAWGYTGGTGGAGTC |
| IgH-UAd-fw10 | Universal Adapter - NNNN GAGGTGCAGSKGGTGGAGTC |
| IgH-UAd-fw11 | Universal Adapter - NNNN GAKGTGCAMCTGGTGGAGTC |
| IgH-UAd-fw12 | Universal Adapter - NNNN GAGGTGAAGCTGATGGARTC |
| IgH-UAd-fw13 | Universal Adapter - NNNN GAGGTGCARCTTGTTGAGTC |
| IgH-UAd-fw14 | Universal Adapter - NNNN GARGTRAAGCTTCTCGAGTC |
| IgH-UAd-fw15 | Universal Adapter - NNNN GAAGTGAARSTTGAGGAGTC |
| IgH-UAd-fw16 | Universal Adapter - NNNN CAGGTTACTCTRAAAGWGTSTG |
| IgH-UAd-fw17 | Universal Adapter - NNNN CAGGTCCAACTVCAGCARCC |
| IgH-UAd-fw18 | Universal Adapter - NNNN GATGTGAACTTGGAAGTGTC |
| IgH-UAd-fw19 | Universal Adapter - NNNN GAGGTGAAGGTCATCGAGTC |
|  |  |
| **IgG Index Reverse** | **TruSeq Adapter Index X (RC) + Diversity region + IgG constant specific region (RC)** |
|  |  |
| IgGall-IdxX-rv | Adapter Index X - NNNN CARKGGATRRRCHGATGGGG |

| **PE - PCR1** |  |
| --- | --- |
|  |  |
| **IgH Extension Forward** | **Extension + VH 5' specific region** |
|  |  |
| IgH-extension-fw1 | CCCTCCTTTAATTCCC GAKGTRMAGCTTCAGGAGTC |
| IgH-extension-fw2 | CCCTCCTTTAATTCCC GAGGTBCAGCTBCAGCAGTC |
| IgH-extension-fw3 | CCCTCCTTTAATTCCC CAGGTGCAGCTGAAGSASTC |
| IgH-extension-fw4 | CCCTCCTTTAATTCCC GAGGTCCARCTGCAACARTC |
| IgH-extension-fw5 | CCCTCCTTTAATTCCC CAGGTYCAGCTBCAGCARTC |
| IgH-extension-fw6 | CCCTCCTTTAATTCCC CAGGTYCARCTGCAGCAGTC |
| IgH-extension-fw7 | CCCTCCTTTAATTCCC CAGGTCCACGTGAAGCAGTC |
| IgH-extension-fw8 | CCCTCCTTTAATTCCC GAGGTGAASSTGGTGGAATC |
| IgH-extension-fw9 | CCCTCCTTTAATTCCC GAVGTGAWGYTGGTGGAGTC |
| IgH-extension-fw10 | CCCTCCTTTAATTCCC GAGGTGCAGSKGGTGGAGTC |
| IgH-extension-fw11 | CCCTCCTTTAATTCCC GAKGTGCAMCTGGTGGAGTC |
| IgH-extension-fw12 | CCCTCCTTTAATTCCC GAGGTGAAGCTGATGGARTC |
| IgH-extension-fw13 | CCCTCCTTTAATTCCC GAGGTGCARCTTGTTGAGTC |
| IgH-extension-fw14 | CCCTCCTTTAATTCCC GARGTRAAGCTTCTCGAGTC |
| IgH-extension-fw15 | CCCTCCTTTAATTCCC GAAGTGAARSTTGAGGAGTC |
| IgH-extension-fw16 | CCCTCCTTTAATTCCC CAGGTTACTCTRAAAGWGTSTG |
| IgH-extension-fw17 | CCCTCCTTTAATTCCC CAGGTCCAACTVCAGCARCC |
| IgH-extension-fw18 | CCCTCCTTTAATTCCC GATGTGAACTTGGAAGTGTC |
| IgH-extension-fw19 | CCCTCCTTTAATTCCC GAGGTGAAGGTCATCGAGTC |
|  |  |
| **IgG Extension Reverse** | **Extension (RC) + IgG constant specific region (RC)** |
|  |  |
| IgGall-extension-rv | GAGGAGAGAGAGAGAG CARKGGATRRRCHGATGGGG |
|  |  |
| **PE - PCR2** |  |
|  |  |
| **IgALL Universal Forward** | **TruSeq Universal Adapter + Diversity region + Extension** |
|  |  |
| IgALL-UAd-fw | Universal Adapter - NNNN CCCTCCTTTAATTCCC |
|  |  |
| **IgALL Index Reverse** | **TruSeq Adapter Index (RC) + Diversity region + Extension (RC)** |
|  |  |
| IgALL-IdxX-rv | Adapter Index X - NNNN GAGGAGAGAGAGAGAG |

**References**

1. Mazor, Y., Van Blarcom, T., Mabry, R., Iverson, B. L. & Georgiou, G. Isolation of engineered, full-length antibodies from libraries expressed in Escherichia coli. *Nat Biotechnol* **25,** 563–565 (2007).
